# Automated detection, delineation and quantification of whole-body bone metastasis using FDG-PET/CT images

**DOI:** 10.1007/s13246-023-01258-z

**Published:** 2023-05-01

**Authors:** R. Nigam, M. Field, G. Harris, M. Barton, M. Carolan, P. Metcalfe, L. Holloway

**Affiliations:** 1 Centre for Medical Radiation Physics, University of Wollongong, Wollongong, NSW 2522 Australia; 2 Ingham Institute for Applied Medical Research, Liverpool, NSW 2170 Australia; 3 Liverpool and Macarthur Cancer Therapy Centre, Liverpool, NSW 2170 Australia; 4 South Western Sydney Clinical Campus, School of Clinical Medicine, University of New South Wales, Sydney, NSW Australia; 5 Illawarra Cancer Care Centre, Wollongong Hospital, Wollongong, NSW 2500 Australia; 6 Institute of Medical Physics, University of Sydney, Camperdown, NSW 2505 Australia; 7 Chris O’Brien Lifehouse, Camperdown, NSW 2050 Australia

**Keywords:** Bone metastases, Oligometastases, Radiotherapy treatment planning, Stereotactic body radiotherapy, Tumour delineation, Multimodality imaging, Image segmentation

## Abstract

Non-small cell lung cancer (NSCLC) patients with the metastatic spread of disease to the bone have high morbidity and mortality. Stereotactic ablative body radiotherapy increases the progression free survival and overall survival of these patients with oligometastases. FDG-PET/CT, a functional imaging technique combining positron emission tomography (PET) with 18 F-fluorodeoxyglucose (FDG) and computer tomography (CT) provides improved staging and identification of treatment response. It is also associated with reduction in size of the radiotherapy tumour volume delineation compared with CT based contouring in radiotherapy, thus allowing for dose escalation to the target volume with lower doses to the surrounding organs at risk. FDG-PET/CT is increasingly being used for the clinical management of NSCLC patients undergoing radiotherapy and has shown high sensitivity and specificity for the detection of bone metastases in these patients. Here, we present a software tool for detection, delineation and quantification of bone metastases using FDG-PET/CT images. The tool extracts standardised uptake values (SUV) from FDG-PET images for auto-segmentation of bone lesions and calculates volume of each lesion and associated mean and maximum SUV. The tool also allows automatic statistical validation of the auto-segmented bone lesions against the manual contours of a radiation oncologist. A retrospective review of FDG-PET/CT scans of more than 30 candidate NSCLC patients was performed and nine patients with one or more metastatic bone lesions were selected for the present study. The SUV threshold prediction model was designed by splitting the cohort of patients into a subset of ‘development’ and ‘validation’ cohorts. The development cohort yielded an optimum SUV threshold of 3.0 for automatic detection of bone metastases using FDG-PET/CT images. The validity of the derived optimum SUV threshold on the validation cohort demonstrated that auto-segmented and manually contoured bone lesions showed strong concordance for volume of bone lesion (*r* = 0.993) and number of detected lesions (*r* = 0.996). The tool has various applications in radiotherapy, including but not limited to studies determining optimum SUV threshold for accurate and standardised delineation of bone lesions and in scientific studies utilising large patient populations for instance for investigation of the number of metastatic lesions that can be treated safety with an ablative dose of radiotherapy without exceeding the normal tissue toxicity.

## Introduction

Advances in radiotherapy have been instrumental in improving local tumour control, alone or in combination with modern multimodality therapies [1]. However, treatment of metastases, which is a major cause of mortality and morbidity, remains a huge challenge. Bone is the third most common site of metastases after lung and liver [2]. Prostate and breast cancer are responsible for the majority of the skeletal metastases (up to 70%), whereas, lung, thyroid, renal cell carcinoma, bladder, and melanoma contribute to nearly 30% of bone metastases [3]. A population-based cohort study of patients with common primary cancer types with bone metastasis showed one-year survival after bone metastasis diagnosis was lowest in patients with lung cancer (10%) and highest in patients with breast cancer (51%) [4]. Another study showed a one-year survival of 37.4% in NSCLC patients with no bone metastasis; 12.1% in patients with bone metastasis but without any skeletal events such as fracture, spinal cord compression; and only 5.1% in patients with both bone metastasis and skeletal events [5].

Historically, bone metastasis is considered incurable, therefore, palliative radiotherapy was offered for symptom control and pain relief. In recent years, numerous clinical studies suggest that the disease in its initial oligometastatic state [6] defined by a small number of metastatic lesions would respond to curative treatment with ablative radiotherapy [7–11]. A phase II randomised trial, called, Stereotactic Ablative Radiotherapy for the Comprehensive Treatment of Oligometastases (SABR-COMET) [12], provides evidence supporting the role of Stereotactic Ablative BodyRadiotherapy (SABR) in oligometastatic-directed therapy (patients with 1–3 metastatic lesions). This study showed median overall survival (OS) was 28 months and progression free survival (PFS) was 6 months for patients given standard palliative care compared to 41 months OS and 12 months PFS in patients treated with palliative plus SABR. In this study cohort the main location of metastases was bone and lung. Recently, a phase III multicentre randomised trial showed ablation of oligometastatic lesions with a single dose of 24 Gy was associated with mitigation of distinct metastatic progression [13]. Based on the encouraging results from randomised trials [11–14], SABR-COMET-10, (phase III trial) [14] aims at application of ablative radiotherapy for patients with 4–10 metastatic deposits. The risk of treatment-related toxicity is likely to increase with increasing number of metastatic lesions treated. One of the main factors for the safe delivery of SABR in such a scenario would be guided by strict adherence to target volume definition with reduced margins and organs at risk (OAR) tolerances to minimise toxicity. In this regard, the radiotoxicity of bone marrow would be a limiting factor in determining the number of metastatic bone lesions that could be safely ablated. Some of the non-standard approaches currently employed in a randomised trial is to define gross tumour volume (GTV) with no clinical target volume (CTV) margin and planning target volume (PTV) margin of no more than 2–5 mm depending on disease, immobilisation, and institutional set-up accuracy [13]. The key to oligometastatic-directed ablative radiotherapy is accurate and standardised target volume delineation to reduce errors and inter-observer variability in GTV delineation and potentially allow for reduction in PTV expansion, thereby decreasing normal tissue toxicity. A consistent GTV delineation will also enable an assessment on the outcomes for that delineation and the potential to adapt the delineation and learn if necessary.

The hybrid imaging technique positron emission tomography (PET) combined with low dose computer tomography (CT) acquired in same session as PET using PET/CT scanner is increasingly being used in radiotherapy for accurate delineation of GTV [15–18]. PET using the radiotracer 18 F-fluorodeoxyglucose (FDG) enables the imaging of glucose metabolism and provides a non-invasive method for detecting and staging NSCLC and identification of metastatic bone lesions due to elevated glucose metabolism in NSCLC [15, 16]. The European Organisation for Research and Treatment of Cancer (EORTC) imaging group evaluated the merits and limitations of all current major imaging modalities for quantification and evaluation of response of bone metastases and 18 F-FDG-PET/CT showed merits in terms of whole-body coverage; reproducibility; ease of use in multicentre trials; outcomes related to PFS/OS; and established response criteria [19]. The RTOG 0515 phase II trial has shown that FDG-PET/CT derived tumour volumes were smaller than those derived by CT alone, resulting in slight reduction in mean lung dose [20]. Bradley et al. [21] have demonstrated a significant reduction in observer variability in target volume delineation using FDG-PET/CT images. Caldwell et al. [22] demonstrated a reduction in inter-observer variability when FDG-PET images were co-registered with planning CT dataset for GTV delineation of advanced NSCLC. Several head and neck cancer studies have shown similar results [23–25]. FDG-PET/CT has also proven superior to CT for detecting bone metastases and infiltration in haematologic malignancies as well as for detecting lytic or mixed osteolytic-osteoblastic metastases in cortical bone [26, 27]. However, determination of cut-off values of SUV for target definition is not a straightforward process. Kwee et al. [28] explains SUV values are affected by various physical, and biological factors such as partial volume and spill over effects, attenuation correction, the reconstruction method and parameters for scanner type, the count noise bias effect, the time between radiotracer injection and imaging, and body size etc. Standardisation of FDG-PET/CT protocols can considerably reduce SUV variability and improve the utility of SUV thresholds among different institutions [29, 30].

Current and future trends in the radiotherapy treatment [7–14], strongly suggest that efficacy of oligometastases-directed ablative radiotherapy would greatly rely on the accuracy and standardisation of the GTV delineation with strict margin control to reduce normal tissue toxicity. This would require mitigation of poor reproducibility and intra-and inter-observer variability in visual determination of GTV, which could be consistently and reproducibly achieved by an automated image segmentation [31–34] tool. By identifying the specific imaging biomarkers of oligometastatic lesions and using those to optimise the segmentation parameters, a standardised and consistent approach, free from observer variability could be used to define a GTV. Moreover, an automated segmentation tool would greatly reduce the time and workload as would be required in any large patient cohort retrospective review. This has motivated the development of a fully automated research and development tool for detection, delineation and quantification of bone metastases using FDG-PET/CT images, with particular application in retrospective studies of oligometastatic-directed radiotherapy of metastatic bone lesions. This tool extracts the anatomical location of bone lesions from the CT dataset and metabolic information from SUV in PET images and then contours GTV in the bone. The automatically contoured GTV could be utilised by computerised treatment planning systems to determine the number of bone lesions that can be safely treated. This work also presents an approach to optimise the parameters for auto-segmentation of bone metastases in a population study.

## Method

Ethics approval was obtained to access FDG-PET/CT scans of patients with stage IV non-small cell lung cancer (NSCLC) diagnosed between 2006 and 2013. The FDG-PET/CT scan was performed with a GE Discovery 710 scanner (GE Healthcare, Waukesha WI). The low dose CT was acquired prior to the PET scan in a 512 × 512 × 311 matrix with a voxel size 1.37 × 1.37 × 3.75 mm and PET images were acquired in 256 × 256 × 311 matrix with voxel size 2.7344 × 2.7344 × 3.27 mm (voxel volume = 0.02445 ml). A retrospective review of FDG-PET/CT scans of more than 30 candidate NSCLC patients was performed and nine patients with one or more metastatic bone lesions were selected for the present study based on visual inspection of co-registered low-dose CT and PET dataset using imaging software MIM (MIM-6.5; MIM Software Inc., Cleveland, OH, USA).

For each dataset, a single radiation oncologist (RO) contoured GTV for each bone metastasis directly onto the diagnostic CT of the FDG-PET/CT using imaging software MIM.

An auto-segmentation algorithm was designed for segmentation of bone lesions using FDG-PET/CT. This algorithm employed thresholding methods [31–33] to combine the metabolic information from FDG-PET, that offers high sensitivity in detection of small, isolated bone lesions [27], and anatomical information from CT. The algorithm was implemented in MATLAB (Mathworks, Natick, MA) version R2018A. The output of the software was visualised using 3D Slicer (http://www.slicer.org [35], a National Institutes of Health–supported open-source platform for medical image informatics, image processing, and three-dimensional visualisation.

The SUV threshold prediction model was designed by splitting the cohort of patients into a subset of ‘development’ and ‘validation’ cohorts. The development cohort was used to determine the optimum SUV threshold for automatic detection of bone metastases. The validity of the derived optimum SUV threshold was tested on the ‘validation’ patient cohort.

### Image segmentation algorithm

**Fig. 1:**
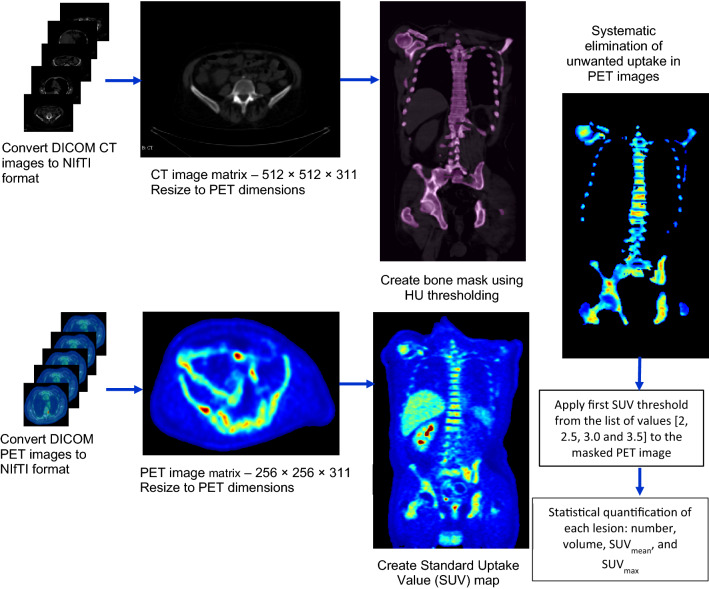
Overview of the segmentation process for bone metastasis

Figure 1 shows the flowchart of the software developed for the detection, delineation and quantification of bone metastases using PET/CT images.

The algorithm comprises of following steps:

PET and CT DICOM files were read and converted into NifTi format [36]. The injected dose of FDG, half-life of FDG, injection time, scan acquisition time and patient weight were extracted from the PET DICOM header. These quantities were used to convert the PET image matrix in bodyweight-SUV. Each slice of the low dose CT image matrix was resized to the dimensions of the PET image matrix using an in-built function ‘imresize’ from the Image Processing Toolbox of Matlab. This function was used to down sample the (x,y) matrix of CT dataset from (512 × 512) to match that of PET matrix of (256 × 256) using bicubic interpolation [37].

The resized CT images were used to create binary bone masks by applying a Hounsfield unit (HU) threshold. A Gaussian smoothing was performed on the bone mask to smooth the edges of the segmented bone, followed by filling holes in the background pixels of the bone mask using the standard in-build MATLAB functions. The optimum HU threshold for bone segmentation was determined using an iterative process. A bone mask was generated by segmenting the bone using CT images by applying a single HU threshold value between 90 and 300 HU. The corresponding bone mask was then overlayed on the CT imageset and the bone structure in CT was visually compared with the segmented bone mask to determine the optimum HU threshold that adequately segmented the entire bony anatomy. The optimisation of HU threshold was performed for all the patients in the development cohort and an average value of 110 HU was determined to include both bone marrow and the cortical bone. The bone mask was applied to the SUV map to eliminate the regions of unwanted uptake, such as bladder, liver, kidney, brain etc.

The metastatic bone lesions were segmented by applying an absolute SUV threshold (SUV-Th) of 2.0, 2.5, 3.0, 3.5, 4 and 6 to the bone-masked-SUV map.

For each SUV-Th, lesion quantification statistics, such as number of lesions, volume, SUV_mean_, SUV_max_, coordinates of SUV_max_ of each auto-segmented lesion were calculated by constraining the SUV map to the binary lesion map to retrieve the SUV information within each detected lesion. The lesion volume was calculated by multiplying number of non-zero voxels in the binary map of the lesion with the voxel volume. SUV_mean_ was computed by taking the summation of SUV of each voxel in the SUV map of the lesion and dividing it by the number of non-zero voxels in the binary map of that lesion. Similarly, the algorithm also calculated lesion statistics of the RO contoured lesions by first converting the DICOM-RTSTRUCT file of manual contours into a label map. The RTSTRUCT is a file in DICOM format that includes the contours for regions of interest commonly relevant to radiotherapy treatment planning.

### Optimisation of the algorithm using the development dataset

For each SUV-Th, regression analysis was completed to compute the coefficient of correlation (concordance) between volume and SUV_mean_ of each auto-segmented and manually contoured lesion (ground truth). To ensure that the manual and auto-contours under test belong to the same anatomical site, auto-contoured lesions were matched to manually contoured lesions using the minimum difference in SUV_max_ and the minimum Euclidean distance between SUV_max_ coordinates. A voxel-by-voxel analysis was performed to find the true positive, true negative, false positive and false negative to determine sensitivity, specificity [38] and dice similarity coefficients (DSC) [39]. Concordance value and DSC were evaluated as a function of SUV-Th to determine the optimum SUV-Th for detection and delineation of bone metastases. Finally, the statistical performance of the binary classification test for selection of optimum SUV-Th was performed using the receiver-operator characteristic (ROC) curve [40].

### Validation of auto-segmentation

The optimised SUV-Th was used for the auto-segmentation of bone metastases in the validation cohort. The algorithm was validated by evaluating the volume and SUV_mean_ concordance, DSC, sensitivity, specificity, and number of lesions, respectively, between auto-segmented and manually contoured bone lesions.

## Results

Table 1 shows the combined demographic of the development and validation patient cohorts. The predominant locations of skeletal metastases in both NSCLC patient cohorts were spine and pelvic girdle.

**Table 1:** Patient demographics (n = 9). All patients have NSCLC with bone metastasis. The statistics are based on the bone lesions contoured by an expert radiation oncologist

	Development cohort	Validation cohort
Mean Age (years)	72.75	65.6
Mean Height (cm)	163.0	162.0
Mean Weight (Kg)	68.75	76.6
Male	1 (11.1%)	2 (22.2%)
Female	3 (33.3%)	3 (33.3%)
Lesion Volume (ml)	0.15–8.9	0.244–74.0
SUV_mean_	2–5.6	2.0–4.8
SUV_max_	2.7–11.1	2.9–8.4
Number of manually contoured lesions	74	52

**Fig. 2:**
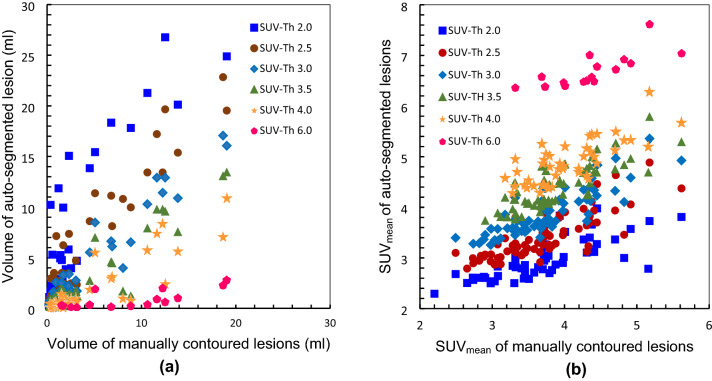
Volume of each auto-segmented lesion using various SUV thresholds versus (**a**) volume of same lesion contoured manually and (**b**) SUV_mean_ for a patient from the development cohort with multiple bone metastases

**Fig. 3:**
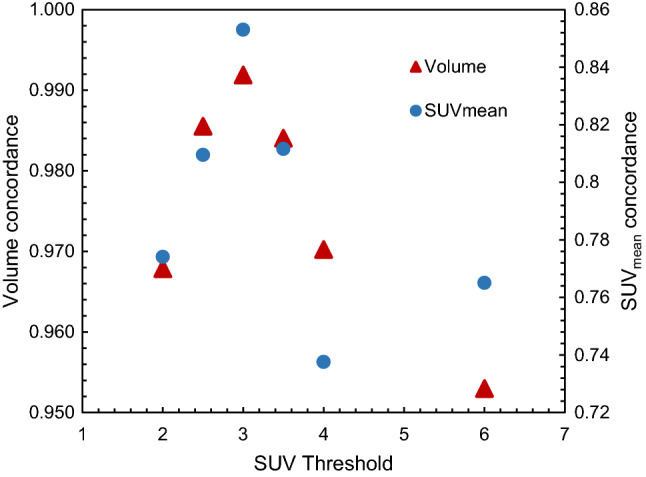
Concordance between manual contours and auto-segmented bone lesion volumes (left) and SUV_mean_ (right), respectively as a function of SUV threshold

Figure 2 shows the results of auto-segmentation of the development cohort for selected SUV-Th values. The influence of SUV-Th could be clearly observed in the volume (Fig. 2a) and SUV_mean_ (Fig. 2b) of each auto-segmented bone lesion. This information when compared to that of the corresponding manually contoured lesion gives an indication of the SUV-Th that results in closest agreement with the ground truth manual contours. SUV-Th of 2.0, 4.0 and 6.0 results in greater difference between the volume and SUV_mean_ of auto-segmented and manual contours. Regression analysis of data in Fig. 2 was performed to estimate the most probable SUV-Th for auto-segmentation that would give the closest agreement to the manual. Figure 3 shows the concordance, *r* between volume and SUV_mean_ of the auto-segmented and manually contoured lesions as a function of SUV-Th. At an absolute threshold of 2.5, 3.0 and 3.5, the coefficient of correlation for volume and SUV_mean_ were found to be greater than 0.98 and 0.8, respectively, suggesting close agreement between auto-segmented and manual contours for any of these thresholds.

**Fig. 4:**
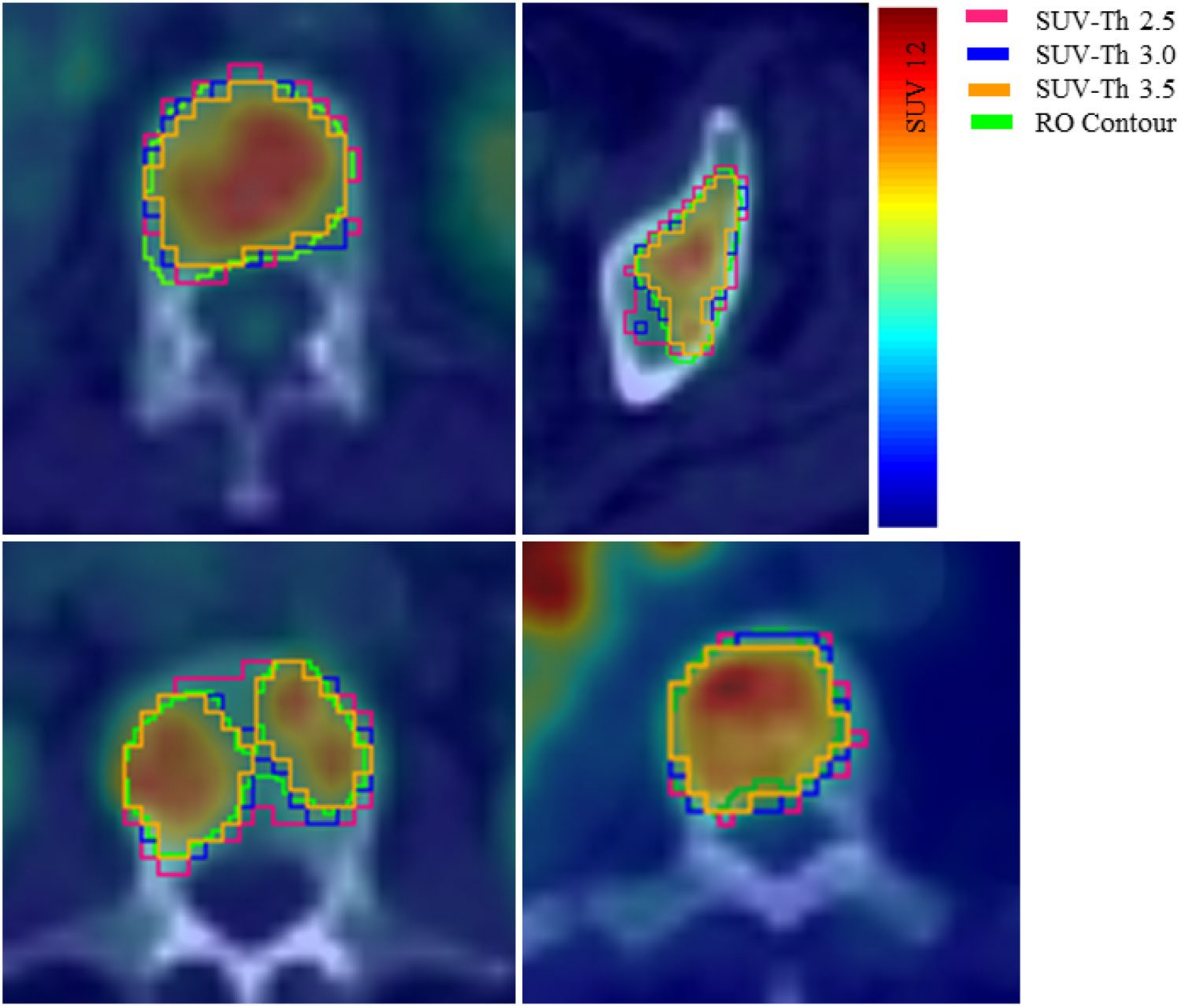
Automatic contouring of high uptake regions of the bone using SUV-Th of 2.5 (pink), 3.0 (blue), 3.5 (orange) and manual contouring by an RO (green)

**Fig. 5:**
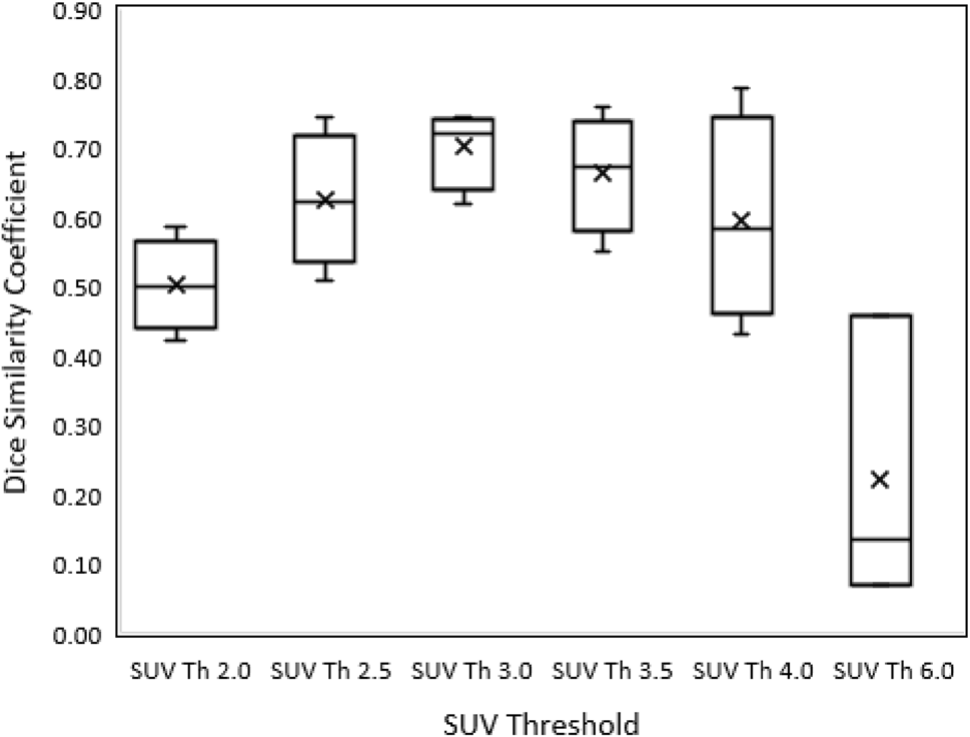
SUV threshold vs. Dice Similarity Coefficient comparing manual contours and auto-segmented contours

**Fig. 6:**
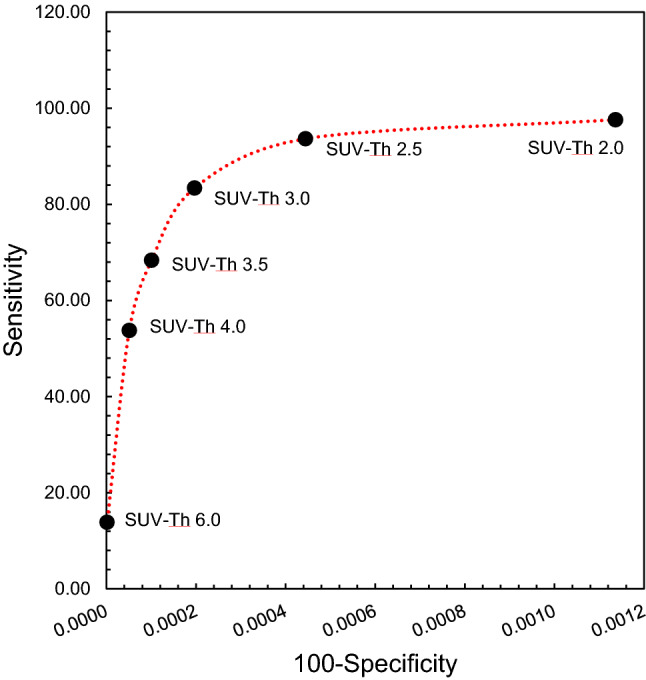
Receiver Operator Characteristics (ROC) curve where each data point represents the true positive rate (sensitivity) and false positive rate (specificity) at SUV thresholds of 2.0, 2.5, 3.0, 3.5, 4, and 6, respectively

Figure 4 shows the axial view of the FDG-PET/CT image with selected osseous lesions from the development cohort, contoured manually as well as with auto-segmentation algorithm using SUV-Th of 2.5, 3.0 and 3.5. Contours corresponding to SUV-Th 3.0 and 3.5 show closer overlap with the manual contours, whereas for SUV-Th of 2.5 a deviation from the manual contour can be observed. Figure 5 shows the algorithm evaluation metric, DSC as a function of SUV-Th. The median DSC increases with increasing SUV-Th, reaching a maximum of 0.72 at SUV-Th of 3.0 and decreasing thereafter. The minimum dispersion in DSC values was observed at SUV-Th of 3.0 whereas large dispersion was found at SUV-Th of 4.0 and higher. This is further corroborated by the strongest linear relationship observed between volume and SUV_mean_ of auto-segmented and manually contoured lesions at SUV-Th of 3.0, with *r* = 0.992 and *r* = 0.853, respectively. The ROC curve in Fig. 6 shows nearly 100% specificity for all the threshold values but highest sensitivity at SUV-Th of 3.0, resulting in maximum sum of sensitivity and specificity. This suggests that SUV-Th of 3.0 is the optimised decision threshold for delineation of bone lesions using FDG-PET/CT images in this study.

**Fig. 7:**
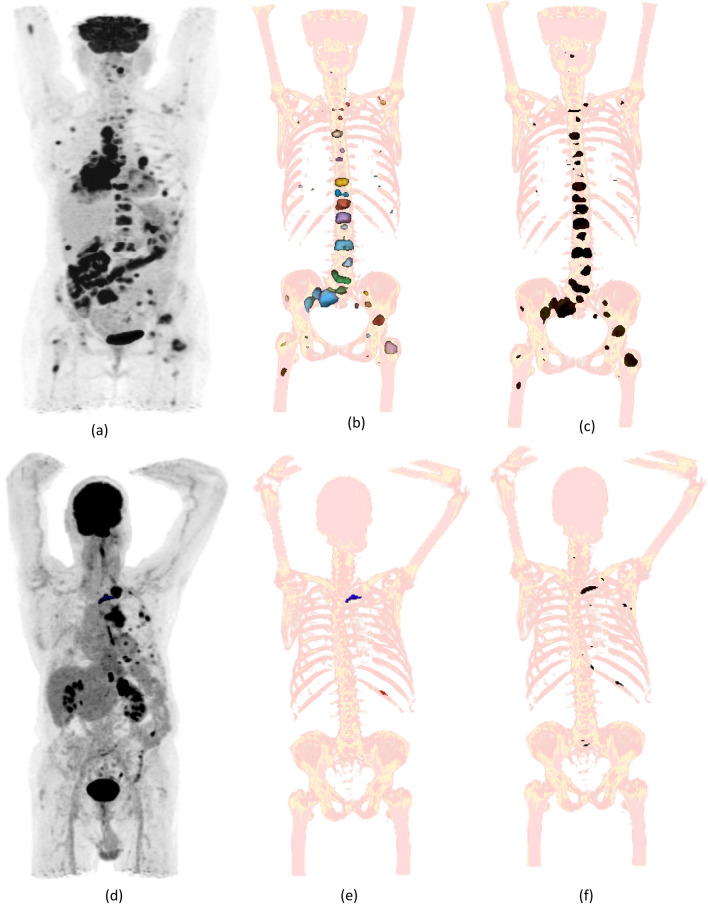
Automatic segmentation of bone metastases using SUV-Th of 3.0 determined from the analysis of validation-cohort. Results shown for two patients, one with widespread diseases and the other with only few bony lesions. **a** and **d** Maximum intensity projection of the PET images of two patients. **b** and **e** multi-coloured bone lesions are the manual contoured lesions by the RO **c** and **f** bone lesions in black are automatically detected using SUV-Th of 3.0

The validation of the auto-segmentation algorithm was performed by segmenting the bone metastases in the ‘validation cohort’ using the optimised SUV-Th of 3.0. Figure 7 provides a visual inspection of auto-segmentation of bone metastases using the optimum SUV-Th of 3.0. Figure 7a and d shows maximum intensity projection (MIP) of a PET image of two patients with distinct metastatic spread of diseases to the bone. Figure 7b and e shows bone lesions contoured by the RO providing visual comparison of results of auto-segmentation of bone metastases in Fig. 7c and f, respectively. The results show excellent performance and efficiency in terms of detection of PET-positive bone lesions.

**Fig. 8:**
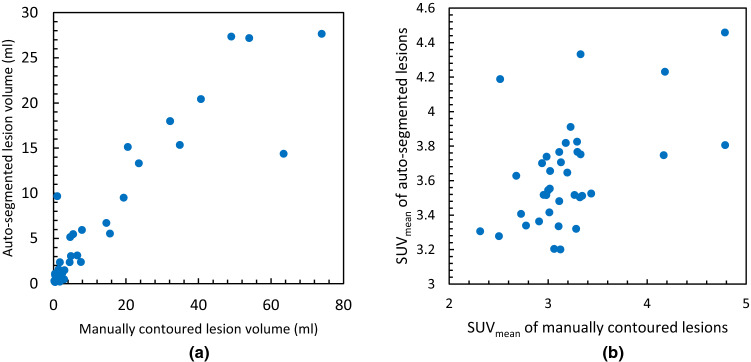
**a** Volume and **b** SUV_mean_ of each auto-segmented lesion using SUV threshold of 3.0 versus volume and SUV_mean_, respectively of same lesions contoured manually. Plot shows the results for entire validation-cohort

The multiple regression analysis of volume and SUV_mean_ of auto-segmented and manually contoured bone lesions of the validation cohort shown in Fig. 8a and b, respectively, yields volume *r* = 0.993 and SUV_mean_ *r* = 0.524. Table 2 presents the validation statistics of auto-segmentation algorithm for each of the individual patients in the validation cohort. Bone metastases was detected with 100% specificity and mean sensitivity of 64.89 ± 12%. The maximum DSC for the validation cohort was calculated to be 0.74 with an average DSC of 0.63 ± 12. A strong concordance of *r* = 0.996 was observed between number of automatically and manually detected lesions in the validation cohort.

**Table 2:** Statistical validation of SUV threshold-based auto-segmentation algorithm using regression analysis, sensitivity, specificity, dice similarity coefficient and number of lesions detected compared to manually contoured lesions

Validation cohort	Volume Concordance	SUV_mean_ Concordance	Sensitivity (%)	Specificity (%)	Dice Similarity Coefficient	Number of manually contoured lesions	Number of auto-segmented lesions
1	–	–	57.1	100	0.71	1	1
2	0.89	0.78	80.2	100	0.74	6	6
3	1.00	1.00	68.1	100	0.69	2	2
4	0.90	1.00	72.8	100	0.42	5	3
5	0.99	0.27	46.3	100	0.60	38	25

## Discussion

In this work, a fully automated segmentation algorithm for detection, delineation and quantification of bone metastases has been presented for potential use in radiotherapy treatment planning for auto-contouring of GTV of bone lesions. The algorithm shows excellent correlation between volume, SUV_mean_ and SUV_max_ of auto-segmented and RO contoured bone lesions, respectively. A high concordance was also observed between the number of lesions detected by SUV-Th based auto-segmentation and RO contours. The results are indicative of high efficiency of the algorithm for detection and segmentation of PET-positive bone volumes. The output of the algorithm is reproducible and observer-independent by nature. A similar SUV thresholding-based auto-segmentation software tool has been developed by Hammes et al. [41], for automated quantification of bone metastasis load in PSMA PET/CT scans for staging and monitoring diseases progression.

The algorithm presented in this study was tested for different SUV-Th values and an optimum SUV-Th of 3.0 was determined based on fitting lesion quantification parameters of auto-segmented FDG-PET-positive volume and manually contoured bone lesions to the multiple regression model. The highest median DSC and least dispersion in DSC values observed at SUV-Th of 3.0 were indicative of maximum overlap between auto-segmented and manual contours for algorithm using 3.0 as the SUV threshold value for auto-segmentation of bone metastases. The ROC curve further confirmed SUV-Th of 3.0 as the optimal binary classifier for detection of bone metastases. The main limitation of this finding is that the optimised value of SUV-Th is based on the single RO contours used as gold standard. An inter-rater comparison, although still not a true gold standard may provide additional evidence on what an acceptable variation is and potentially substantiate the current work further.

**Fig. 9:**
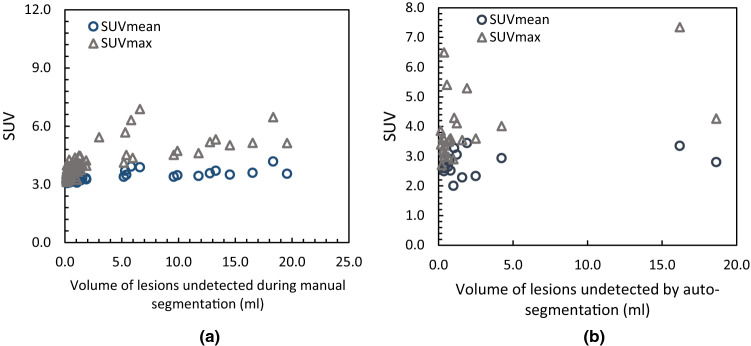
Combining the results of auto-segmentation of bone lesions in validation-cohort using SUV-Th of 3.0, plot (**a**) shows lesions segmented by the auto-segmentation algorithm but remaining undetected during manual contouring; and (**b**) lesions manually contoured but remaining undetected by the auto-segmentation algorithm. Each data point is the lesion represented by its volume, SUV_mean_ and SUV_max_

Whilst, the algorithm demonstrates excellent performance, some of the lesions contoured by the RO remained undetected by the auto-segmentation algorithm as shown in Fig. 9. To understand the potential deficiency in the auto-segmentation algorithm, as a part of optimisation process, the PET-positive volumes that were misclassified as true positive (Fig. 9a) and true negative (Fig. 9b) by the algorithm were presented. Each of the data points in both the graphs are the PET-positive volumes from the validation cohort, represented by the corresponding SUV_mean_ and SUV_max_ values. Figure 9a shows that PET-positive uptake undetected by the visual inspection ranged from SUV_mean_ of 3.0 to 6.0. In the absence of inter-rater comparison, it could not be concluded whether the missing manual contours were metastatic bone lesions or benign anomalies, casting doubt on the classification of auto-segmented voxels. However, concordance between the majority of auto-segmented and manual PET-positive volumes with SUV_mean_ greater than 3.0 does seem to suggest a potential intra-observer variability. On the contrary, the PET-positive volume contoured by the RO but not detected by the auto-segmented at SUV-Th 3.0 have an SUV_mean_ of less than 3.0 and due to the nature of the algorithm would remain undetected. Some of the small RO contoured volumes with SUV_mean_ and SUV_max_ greater than 3.0 remained undetected by the algorithm. This was investigated by registering on the PET/CT dataset the associated bone mask and overlaying RO contours and auto-segmented contours on this dataset. The results displayed that the PET-positive volumes undetected by the algorithm resided outside the auto-segmented bone, hence remained undetected by the algorithm. Since the algorithm only searches for the PET-positive volume within the segmented bone, a potential deficiency exists in selecting a universal HU threshold for bone segmentation of the entire population. The segmented bone region comprises of low-density bone marrow and high-density cortical bone which in a CT image is manifested as lower HU value for bone marrow compared to cortical bone due to differences in their linear attenuation coefficients. The differences in the bone densities of individual patients further results in the variability of HU values. If the auto-segmentation algorithm is to be used in the clinical setting, the HU threshold for bone segmentation should be kept at a reasonably low value to incorporate both low density bone marrow and high-density cortical bone in the segmented bone volume.

The auto-segmentation algorithm has been designed to detect the lesions confined to the bony anatomy of the patient. Any bone lesion infiltrating into the surrounding normal tissue would remain undetected. This limitation could be addressed by extending the absolute thresholding algorithm to a region growing algorithm [42], where the final classification of PET-positive voxels would be performed on the entire PET data matrix, unlike SUV-Th algorithm which classifies the voxels only on the bone-masked PET data. In this case, the SUV threshold-based segmentation algorithm presented in this work could be used as a starting point for region-growing algorithm to perform the first step of auto-segmentation of PET-positive volumes. In the next step, the coordinates of SUV_max_ of each of segmented volumes could be used as a seed to search the neighboring PET-positive voxels in the PET data matrix (SUV map) resulting in detection of extraosseous extension of the diseases. The algorithm may result in false-positive classification of voxels in cases where lesion in bone shares a boundary with high FDG uptake organs such as liver, kidney, bladder etc. Nevertheless, this method has potential to reduce the dependency of HU threshold on the performance of the algorithm.

The use of PET/CT images in current radiotherapy treatment planning incorporates rigid registration of the low dose CT component of the PET/CT image with the high-quality planning CT images. A visual assessment of high uptake regions in co-registered PET image guides the delineation of target volume on the planning CT images. The potential failure to contour the PET-positive volume in its entirety is compensated by increased margins to define clinical target volume (CTV) and planning target volume (PTV). For ablative radiotherapy techniques, such as SABR, reduction in margins to within setup and machine tolerance is crucial to avoid the ablation of normal tissue. The segmentation tool presented here would provide automated quantification of GTV volume based on PET/CT images. This opens the scope of this algorithm to be utilised in delineation of tumour sub-volumes, called biological tumour volume (BTV), which has become a point of interest in biologically oriented radiotherapy [43]. The idea is that by selectively ablating BTV while decreasing the dose to more susceptible regions, local tumour control rates could increase without increased side effects [44]. However, there are number of uncertainties in BTV/GTV contouring related to poor spatial resolution of PET images as well as the internal and external patient motion. In this study, the use of image dataset from PET/CT scanner instead of a PET-only scanner minimizes these uncertainties. In a PET/CT scanner, the high-resolution CT images and inherently low-resolution PET images are taken in the same session under reproducible patient setup resulting in reduced registration error between the two image sets and reduced uncertainties associated with patient external motion, consequently resulting in more accurate localisation of the tumour volume. In terms of internal motion, the, bone is a robust structure and not subjected to internal motion unless lesion is in the rib, which may be impacted by respiratory motion and may require respiratory-gated PET/CT-imaging [45]. To further account for any patient motion-related systematic error in target localisation, prior to selecting the patient’s PET/CT dataset for the study, a retrospective review was performed to identify any potential motion-related or otherwise, considering a mismatch between the two datasets using MiM software. An additional check was performed at the time of validation of the auto-segmentation tool, where output files, i.e., bone mask, SUV map and the auto-segmented bone lesions were overlaid on input PET/CT dataset and visualized using 3D Slicer software to ensure the accuracy of geometrical match.

Another limitation of an SUV-Th based algorithm is the uncertainty in SUV values which is associated with injected FDG dose, the time between radiotracer injection and imaging, body size and weight and scanner type [28]. Based on several studies, a maximum SUV threshold of 2.5 has long been used to separate benign from malignant lesions [46, 47]. There are very limited studies in the literature that have evaluated the optimum SUV-Th for GTV delineation. Yu et al. [48] evaluated the threshold value of SUV based on FDG-PET/CT images that generates the best volumetric match to the pathologic GTV for NSCLC. Their results showed 31 ± 11% of SUV_max_ and an absolute SUV threshold of 3.0 ± 1.6 were the most appropriate cut-off values for target volume definition on FDG-PET/CT images for NSCLC. Phantom studies have shown an optimum SUV-Th of 40–50% SUV_max_ [33]. Standardisation of FDG-PET/CT protocols can considerably reduce SUV variability and improve the utility of SUV thresholds among different institutions [29, 30]. In this work, a single institution study using a departmental FDG-PET/CT imaging protocol was employed for the imaging of patients in the development and validation cohort, resulting in an unambiguous determination of SUV-Th of 3.0 as optimal threshold for bone metastases in stage 4 NSCLC patients, corroborating the standardised imaging approach advocated by Kwee et al. [28]. Nevertheless, further validation studies and clinical trials are required to investigate the above-mentioned uncertainties in GTV contouring using PET/CT images before PET/CT -based radiotherapy treatment planning can be used in routine clinical practice. The validation module of the auto-segmentation tool presented in this paper is designed to provide a research framework for detailed big data analysis of a large cohort of PET/CT images to investigate the correlation between SUV and various patient-, scanner- and protocol-related uncertainties, respectively, to determine an optimum SUV for accurate delineation of GTV.

The algorithm presented here is designed to classify the voxel as true positive if the voxel uptake value is higher than the threshold, the algorithm is unable to differentiate between a malignant tumour and inflammation or fracture in bone that results in similar uptakes as a malignant tumour in the PET images. In future, the algorithm could be modified to extract radiomic features to distinguish between malignant and benign features. The SABR-COMET trial outcomes suggest identification of biomarkers of oligometastasis can help select those patients who are most likely to benefit from oligometastatic-directed therapy [14]. Until then the visual assessment of the auto-segmentation of PET-positive volume by the RO, based on the clinical experience would be the best way forward, if the auto-segmentation tool is to be used in clinical radiotherapy planning.

Furthermore, the tool has various other applications in scientific research such as a quick retrospective review and quantification of metastatic bone lesions in FDG-PET/CT images of a large patient population, free from intra-and inter-observer variability. Provided the segmentation parameters can be standardised, this tool can be used in the clinical setting for detection and segmentation of bone metastasis, which would help in staging and progression of the disease. With a standardised automated process, this tool would help reduce the clinical workload. The tool can be modified to incorporate machine learning methods for quantification of radiomic biomarkers [49], for stratification of tumour histology, tumour stages and clinical outcomes or as suggested in [14], identify biomarkers of oligometastasis that can help select those patients who are most likely to benefit from oligometastatic-directed ablative radiotherapy. Moreover, this tool could be used when considering large datasets where consistency is desired as the Australian Computer-Assisted Theragnostics (AusCAT) network for radiation oncology data extraction, reporting and federated learning on datasets distributed across clinics [50].

## Conclusion

A fully automated, robust, reproducible, and user-independent auto-segmentation tool has been presented for detection, delineation and quantification of PET-positive bone lesions using FDG-PET/CT images for use in radiotherapy. The auto-contoured metastatic bone lesions show a strong agreement with the manual contours of radiation oncologist. The software tool provides flexibility in terms of optimisation of the threshold parameters for decision on classification of the disease as a true-positive. The tool enables this by performing an automated statistical validation of the auto-contours against the ground truth. The optimised threshold could then be fed-back to the software to enable reliable auto-segmentation of bone metastases in a large patient population.
